# Catch 22, Giant Congenital Melanocytic Nevus in a Florid Keloid Former

**Published:** 2014-05-09

**Authors:** Abdulrasheed Ibrahim, Malachy E. Asuku

**Affiliations:** Division of Plastic and Reconstructive Surgery, Department of Surgery, Ahmadu Bello University Teaching Hospital, Zaria, Nigeria

**Keywords:** giant congenital melanocytic nevus, disfiguring, malignant transformation, keloids, recurrence

**Figure:**
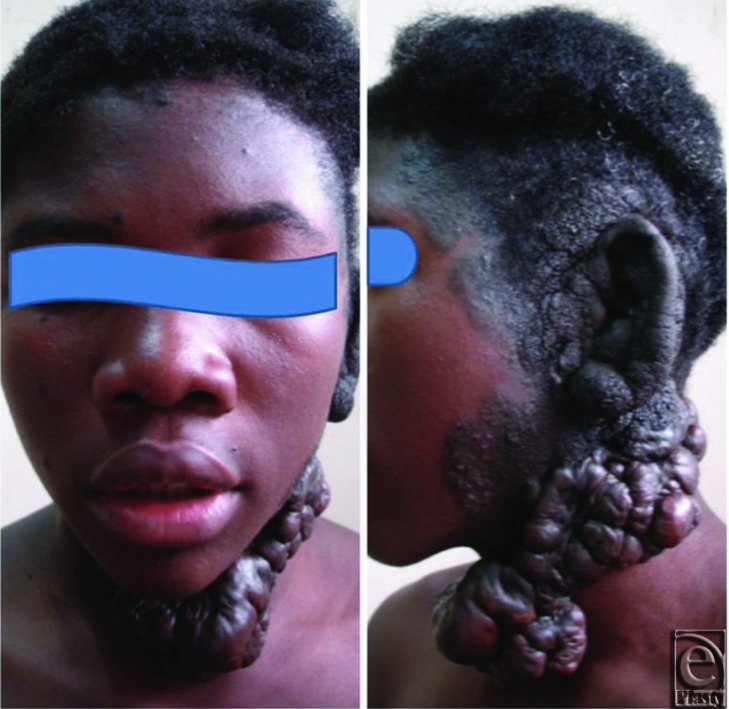


## DESCRIPTION

A 16-year-old adolescent boy presented to our clinic with an extensive hyperpigmented lesion on the left side of the scalp, neck, and face involving the entire left ear. The lesion was present at birth, increasing proportionally in size and developing nodules and coarse hair follicles as the patient advanced in age. Excision of part of the lesion on the neck in a peripheral hospital 4 years earlier resulted in a hard, nodular, and itchy lesion that has continued to increase in size beyond the original surgical scar.

## QUESTIONS

1. **Discuss the diagnosis of the primary lesion.**
2. **Discuss the diagnosis of the secondary lesion.**
3. **What are the differential diagnoses of the primary lesion?**
4. **What are the goals of treatment of the primary lesion?**

## DISCUSSION

This rare and disfiguring skin lesion is a giant congenital melanocytic nevus (GCMN). It is characterized by variability in size and shape, dark pigmentation, and verrucous hair-bearing surfaces.^1^^,^^2^ Although same in morphology and histology, congenital melanotic nevi are classified on the basis of surface area into giant, medium, and small lesions. GCMN is described as one measuring 20 cm or greater in diameter in an adult or predicted to reach 20 cm in diameter by adulthood. The incidence of congenital melanotic lesion is put at 1% and approximately 0.002% for the giant variety. Aside from the psychosocial burden of disfigurement, the lesion is not known to cause any constitutional disturbance. Highest predilection is for the trunk where it is described as a bathing trunk nevus. Involvement of the head and neck region is relatively uncommon. The risk of malignant transformation into a melanoma has been put at about 2% to 10%, justifying concerted efforts at early reduction of nevocytic density, particularly as 50% of transformations occur in the first 3 years of life. The risk is highest with multiple, large lesions in younger patients.^3^^,^^4^

The secondary lesion on the neck resulting from surgical excision is a keloid, resulting from a fibroproliferative disorder of wound healing with unsavory reputation for recurrence. Occurring in genetically predisposed individuals, the etiopathogenesis of keloids is as uncertain as is its protean manifestations. Keloid formation has remained the bane of plastic surgical practice in sub-Saharan Africa. The bedrock of management has included intramarginal excision, intralesional injection of steroids, and radiotherapy, either individually or in any combination, yet control rather than cure remains the measure of success.

The differential diagnosis of GCMN in the early stages includes other epidermal pigmented skin lesions such as hemangiomas, nevus psoriasis, verruca vulgaris, inflammatory linear verrucous epidermal nevus, and hamartomas. In its advanced stages, lesions such as cylindroma presenting as the turban tumor, malignant melanoma, pigmented basal cell carcinoma, dermatofibroma, and dermatofibrosarcoma deserve due consideration.

The goals of treatment of GCMN are 2-fold: reducing nevocytic density albeit risk of malignancy and providing cosmetic relief for the disfigurement. Full-thickness surgical excision with reconstruction of the resultant defect is therefore the mainstay of treatment. Lesions in the head and neck region often constitute significant reconstructive challenges requiring the deployment of the full armamentarium in the plastic surgeon's arsenal—from skin grafts through tissue expansion to microvascular free flap reconstruction.^5^ Laser fulguration has been reported, but the contention has been the possibility of residual nevus cells with malignant potentials in the depth of the lesion. The extensive size of the lesions coupled with the desire for early intervention has made serial excisions and closure a common recourse. Suffice to mention that management must be individualized; however, the importance of lifelong follow-up and surveillance cannot be overemphasized.^5^^,^^6^

Experience with hypertrophic scarring and keloid formation continues to play a major role in the choice of reconstructive techniques in our patient population.^7^^,^^8^ Aside from contributing to the patient's disfigurement, the keloid was associated with pain, itching, and drainage, making it a priority on the inventory of needs. Marginal excision with split-thickness skin graft closure and serial injections of triamcinolone acetonide started 2 weeks after surgery were successful in controlling the keloid. We have always been skeptical of radiation to the head and neck region for a benign disease in relatively young patients.^8^ The patient, however, declined further excisions of the giant melanocytic nevus for fear of keloids on his face, a genuine concern by all means!
